# Assessing the Spread of the Sport of Padel and the Prevalence and Causes of Injuries Among Padel Players

**DOI:** 10.3390/healthcare13040367

**Published:** 2025-02-10

**Authors:** Ayman Alhammad, Husam Almalki, Hussain Ghulam, Renad Al-harbi, Samia Al-harbi, Shaima Al-shareif, Omar Althomali, Redha Taiar

**Affiliations:** 1 Department of Physiotherapy, College of Medical Rehabilitation Sciences, Taibah University, Al-Madinah Al-Munawarrah 41477, Saudi Arabia; renod2003@hotmail.com (R.A.-h.); ptsamia345@gmail.com (S.A.-h.); shaima1234840@gmail.com (S.A.-s.); 2 Department of Health Rehabilitation, College of Applied Medical Sciences at Shaqra, Shaqra University, Shaqra 11961, Saudi Arabia; halmaliki@su.edu.sa; 3 Department of Physical Therapy, Faculty of Applied Medical Science, Najran University, Najran 55461, Saudi Arabia; hsghulam@nu.edu.sa; 4 Department of Physical Therapy, College of Applied Sciences, University of Ha’il, Ha’il 81422, Saudi Arabia; o.althomali@uoh.edu.sa; 5 Department of Sport Science, MATériaux et Ingénierie Mécanique (MATIM), Université de Reims Champagne Ardenne, CEDEX 2, 51687 Reims, France; redha.taiar@univ-reims.fr

**Keywords:** padel, injuries, musculoskeletal pain, prevalence, risk factors, injury prevention, rehabilitation, player well-being

## Abstract

Objectives: To study the prevalence of injuries among padel players in Madinah and investigate potential causes. This study makes an attempt to add to the gaps in the literature regarding injury risks and preventive strategies in this fast-growing sport. Methods: Retrospective cross-sectional study on 305 padel players who come from Madinah, consisting of 193 men and 112 women aged 18–40 years. Data were collected using an online Google Forms questionnaire, including descriptive statistics and non-parametric tests, among which the chi-square test was performed, aiming for the assessment of demographic and injury-related variables. Results: There were significantly different incidences of injuries with regard to gender at the *p* = 0.001 level. A 44.6% prevalence was recorded among women, while men had a prevalence of 8.2%. With respect to the severity of injuries from moderate to severe, there are higher percentages in women that comprised 40.4% and 5.6%, respectively. The most frequent types of upper body injuries among women were ligament sprains and muscle strains. Stress and poor warm-up practices were some of the lifestyle factors identified to increase the risk of sustaining an injury. Conclusions: This study highlights gender-specific injury patterns among padel players in Madinah, emphasizing the need for targeted injury prevention programs, including structured warm-ups and strength training. The findings contribute valuable insights for enhancing player safety and aligning with public health objectives under Saudi Vision 2030.

## 1. Introduction

Padel is a racket game that is played on a 20-meter long and 10-meter wide court surrounded by walls and divided by a central net [1]. During the last years, padel has become so popular worldwide that millions of players actively participate in the sport [2]. This surge in interest is also evident in Madinah, Saudi Arabia, where padel has experienced remarkable growth. According to the Saudi Padel Committee (2024), there are approximately 13,149 active players and 19 padel courts in Madinah [3]. These numbers underscore the growing enthusiasm for padel within the local community.

Moreover, padel has numerous health benefits that include an improvement in the quality of life, a lower body fat percentage, and good cardiovascular fitness [4], which is parallel to Saudi Vision 2030, which encourages different sports in the kingdom. It is also in line with the nation’s strategic goals in raising physical activity and community well-being.

Research on padel has expanded significantly in recent years. Muñoz et al., in 2023, outlined the prevalence of occupational injuries among padel coaches, and the development of injury prevention strategies was considered paramount for the sport [5]. Also, Sánchez-Alcaraz et al., in another systematic review conducted in 2023, highlighted the rapid growth of the sport and the need for optimization of performance with mitigation of injuries [6]. Furthermore, Martín-Miguel et al. (2024) explored the physiological and anthropometric parameters of padel players, providing valuable insights into the demands of the sport [7]. Performance analysis by Martín-Miguel et al. (2023) also emphasized the specific movements and risks associated with padel, underscoring the need for targeted interventions [8].

Despite these benefits, padel carries an inherent risk of injury. A systematic review by Dahmen et al. (2023) calculated an injury incidence of 3 per 1000 training hours and 8 per 1000 practice matches [9]. Muscle strains and tendinitis are common; the elbow is the most commonly involved region, followed by the lower back, knee, and shoulder. Several factors contribute to these injuries, including player strength, racket control, and equipment, such as shoes and court surface conditions [10].

Racket sports like padel place unique demands on the body, leading to specific injury patterns. Compared to other racket sports, such as tennis or badminton, padel involves a higher frequency of lateral movements and quick volleys, increasing the risk of lower limb injuries [5,6]. Age plays a critical role; younger players often experience overuse injuries due to high-intensity training schedules, while older players face risks related to slower recovery rates and cumulative strain over time [7]. Gender differences are also evident in injury patterns. Women are more prone to ligament sprains due to greater joint laxity and hormonal influences, while men may have a higher prevalence of muscular injuries owing to differences in muscle mass and biomechanical forces during play [8,10].

Injuries among padel players vary between lower and upper limbs. Lower limb injuries, such as Achilles tendon strains and knee joint issues, are prevalent, with one study identifying the Achilles tendon as the most affected area [11]. Conversely, upper limb injuries, particularly elbow injuries, dominate in other studies, highlighting lateral elbow tendinopathy as a frequent issue [12]. Such findings underscore the importance of addressing both lower and upper limb injury risks.

Several studies have investigated the risk factors associated with padel injuries [13,14,15]. Equipment choices, such as racket type and weight and playing habits, including training volume and session duration, significantly influence injury risk [13]. Demographic factors, including age and gender, have also been studied, with findings often showing variability in their impact on injury risk [14]. While some research emphasizes the role of footwear and playing conditions, other studies highlight the need for comprehensive studies to generalize findings across different environments and populations [15].

Structured warm-up and injury prevention programs are important in reducing the risks of injury. Warm-up interventions, including balance exercises, dynamic stretching, and sport-specific routines, have been reported to significantly reduce injury rates [16]. Neuromuscular training programs, which incorporate balance, core stability, and strength exercises, further enhance physical readiness and minimize injury risks [17]. However, the adoption of these practices among recreational players remains inconsistent, highlighting an area for improvement.

Despite the growing body of research on padel injuries, there is a notable lack of studies focusing on players in Madinah. This gap limits the understanding of region-specific challenges and risk factors. Additionally, there is limited information on gender-specific injury patterns and the effectiveness of preventive measures in this demographic.

This study aims to address these gaps by studying the spread of padel and the incidence of injuries and their possible causes among padel players in Madinah. The findings are expected to guide injury prevention strategies and promote safer participation in this rapidly growing sport. 

## 2. Methodology

The current investigation was a cross-sectional design undertaken with the aim of analyzing characteristics and injury prevalence and their associated risk factors among padel players in Madinah. The cross-sectional design allowed data collection to be taken from a defined population at one point in time, therefore clearly providing a snapshot of the players’ demographics, playing habits, and injury experiences.

### 2.1. Ethical Considerations

The study was approved by the Taibah University Ethics Committee (Approval Number: CMR-PT-2024-19). The participants were informed about the purpose of the research, the voluntary nature of participation, and measures regarding data confidentiality. The participants gave electronic consent before answering the questionnaire. Data anonymization was performed, and all files were kept password-protected in order to maintain the security and confidentiality of responses according to ethical requirements.

### 2.2. Participants and Sampling

The target population was padel players in Madinah who were aged between 18 and 40 years. From these, a total sample of 305 participants took part in this study; 193 male and 112 female respondents were identified through a convenience sampling approach. Recruitment was conducted physically at every local padel club and via social networks like Instagram and WhatsApp for padel player groups as a means of ascertaining broad reach with an appropriate sample size.

The age range was chosen to include active adult players, reflecting the most engaged demographic in padel participation [18]. While convenience sampling may limit generalizability, it was deemed appropriate given the exploratory nature of the study.

### 2.3. Questionnaire Design

The structured questionnaire was designed to capture relevant data and consisted of four main sections:

- Demographics: Age, gender, and duration of padel practice (in months).
- Playing Habits and Goals: Weekly playing padel, session time (hours), self-classification as a player, and personal goals for playing padel.
- Injury and Pain Assessment: History and severity of injuries, frequency of musculoskeletal pain, location of the pain, and the impact of injuries.
- Prevention Practices: Warm-up routines, use of special shoes, awareness of injury risks, and any guidance received on injury prevention.

The questionnaire was developed based on the existing literature and refined through an expert review process, involving a physical therapy professor, a consultant in sports injuries, and a researcher specializing in sports sciences. Specific feedback included rephrasing ambiguous questions, adding items related to warm-up practices, and improving the logical sequence of sections [19].

### 2.4. Pilot Testing and Reliability

A total of 15 padel players in Madinah piloted the questionnaire to check its clarity, relevance, and usability. The reliability of this study was checked using Cronbach’s alpha and obtained a coefficient of 0.87, indicating high internal consistency [20]. This ascertained that the questions measured the intended constructs well.

### 2.5. Data Collection

Data collection spanned three months, utilizing both physical distribution at padel clubs and online dissemination via social media platforms. This dual approach ensured the inclusion of diverse participants across various skill levels and playing frequencies.

### 2.6. Statistical Analysis

Statistical analysis was conducted using IBM SPSS (version 26, IBM Corp., Armonk, NY, USA). Descriptive statistics summarized demographic data, while inferential statistics were employed to explore associations and differences between variables. The chi-square test was used to analyze categorical variables, such as injury prevalence and gender differences. This test is appropriate for determining relationships in larger datasets and is particularly effective when dealing with nominal data. 

The statistical significance for all analyses was set as *p* < 0.05. Our study adopted a cross-sectional design in order to explore the characteristics and injuries of padel players in Madinah. Data were collected through a structured questionnaire that was aimed at eliciting information on relevant variables of the players’ personal details, history of injuries, and other related factors.

## 3. Result

### 3.1. Participant Demographics

Women showed a higher average age, with 27.82 ± 6.04 years, as compared with men, who had a mean of 24.36 ± 5.30 years (Table 1). Thus, the overall average age for the participants considering both genders was 26.55 ± 6.01 years. Time practicing padel differed between genders. χ^2^ = 0, *p* < 0.001. Women had been practicing padel for a mean duration of 16.60 ± 8.21 months, whereas men reported a mean duration of 10.75 ± 6.45 months. On average, participants had practiced padel for 14.45 ± 8.10 months.

**Figure 1:**
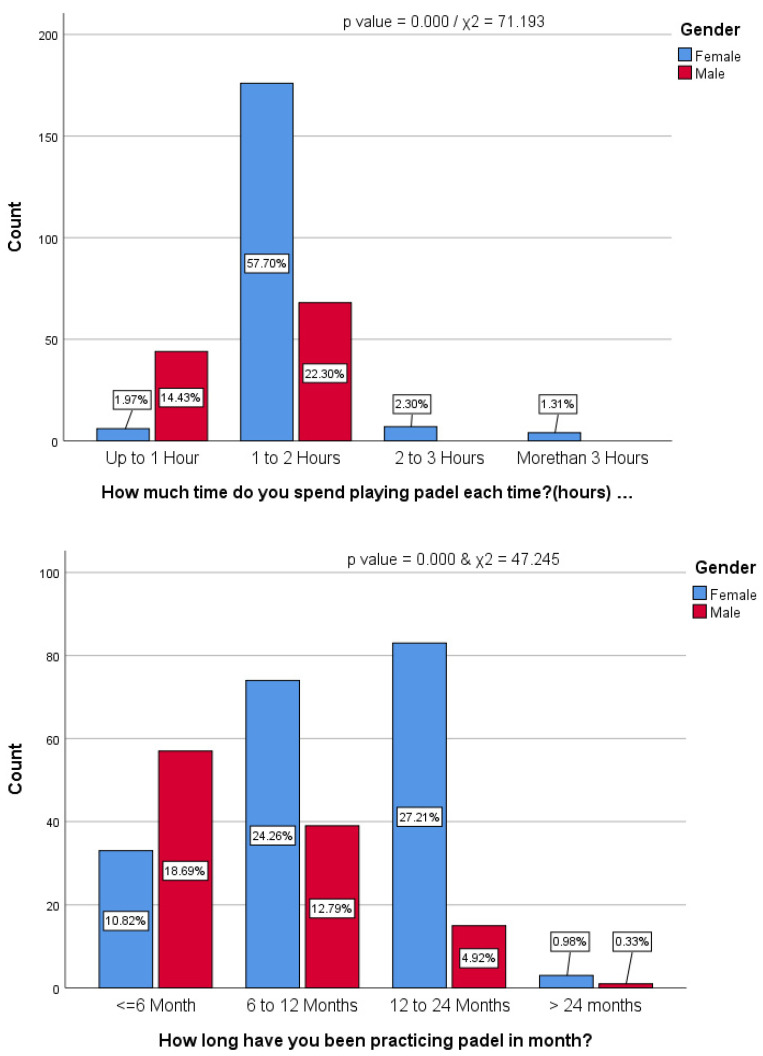
Practicing and playing timing.

Figure 1 presents the average of padel practice and playing time per session for both men and women. The duration of playing padel per session did not indicate any significant difference between men and women; χ^2^ = 0.188, *p* > 0.05. Among the female players, 1.87 ± 0.57 hours is the average time spent playing padel, while males spent around 1.35 ± 0.32 hours. The average duration of playing padel was 1.68 ± 0.55 hours.

### 3.2. Equipment Usage and Satisfaction

There were significant differences between men and women players with regard to special shoes for playing padel; χ^2^ = 0.010, *p* < 0.05. The percentage of respondents using special shoes was higher in the case of female respondents, 41.6%, compared to male respondents, who constituted 18.7%, while the overall utilization rate was 60.3%.

### 3.3. Satisfaction Levels

No significant gender differences were observed in satisfaction levels with the padel experience (χ^2^ = 0.239, *p* > 0.05). Among women, 41.3% reported being satisfied, while 21.0% reported being sometimes satisfied. Among men, 26.6% reported being satisfied, and 11.8% reported being sometimes satisfied. Overall, 62.3% of all respondents reported being satisfied with their padel experience.

### 3.4. Frequency of Playing Padel

Table 2 presents the orientation factors of padel game participation, including the frequency of play, professional level, injury incidence, and players’ goals for practicing padel. The frequency of playing padel per week varied significantly between genders (χ^2^ = 49.424, *p* < 0.001). Many female respondents played 1–3 times per week (13.8% once, 18.0% twice, and 18.0% three times), while male respondents were more evenly distributed over the frequency categories with 22.0% playing once, 7.2% twice, and 5.6% three times per week. A relatively high percentage of male respondents played 4–7 times per week compared to their female counterparts.

### 3.5. Professional Levels

There were significant differences between men and women players regarding professional levels (χ^2^ = 51.308, *p* < 0.001). Men who identified themselves as amateur, intermediate, and professional within the game were 23.3%, 11.1%, and 2.3%, respectively. while women who identified themselves as amateur, intermediate, and professional within the padel game were 14.1%, 39.3%, and 9.8%, respectively. These results show that men are more represented within the amateur level and within the intermediate and professional level.

### 3.6. Injury Incidence and Severity

Table 3: Comparison of injury severity, types, pain locations, musculoskeletal pain experiences, and awareness of injury risks among female and male padel players. There were significant gender differences in the incidence of injuries while playing padel (χ^2^ = 65.915, *p* < 0.001), with 44.6% of women compared to 8.2% of men experiencing injuries during playing. The overall difference in severity of the injuries in men and women was highly significant, with χ^2^ = 5.640 and a *p*-value of 0.060. Among women, 39.1% reported having minor injuries, followed by moderate injuries at 40.4%, while 5.6% reported serious injuries. The proportions of reported male injured patients having minor and moderate injury were found to be 10.6% and 4.3%, respectively; none of the male patients sustained serious injury.

### 3.7. Types and Locations of Pain

The types of injury suffered by the padel players were strongly related to gender. χ^2^ = 72.502; *p* < 0.001. The majority of them complained of ligament sprain injuries for women, 10.1%, and men, 3.0%, followed by muscle strains for women, 12.1%, and for men, 1.3%.

Chi-square distribution by gender in the pain location after playing padel is statistically significant: χ^2^ = 10.254, *p* = 0.006. Women: lower body—19.7%, upper body—42.2%, both—2.4%; men: lower body pain was reported by 5.1%, upper body by 29.6%, and pain in both locations was declared by 1.0% of the respondents.

### 3.8. Pain Frequency After Playing and Management

There was a statistically significant difference in the frequency of musculoskeletal pain: χ^2^ = 16.841, *p* = 0.001. Specifically, 29.5% of women reported pain sometimes, while 4.9% always experienced pain. Among men, 11.5% reported pain sometimes and 2.6% reported pain always.

The most important strategies to manage musculoskeletal injuries included rest and relaxation techniques for women and men, respectively, adopted by 34.2% and 29.5%, respectively. Ice therapy was adopted by 4.2% of women and 6.3% of men. Rest and ice therapy combined was adopted by 7.8 and 2.7%, respectively. A very few sought medical advice.

### 3.9. Lifestyle Factors Contributing to Injury Risk

Several lifestyle factors were linked to increased injury risk. Stress levels were the most significant factor, reported by 94.8% of respondents, followed by diet and nutrition (72.8%) and physical activity levels (75.7%). Previous injuries (46.9%), poor sleep quality (50.2%), and smoking (71.8%) were also notable contributors.

### 3.10. Impact of Pain on Daily Activities

Table 4 summarizes the gender-based comparison of musculoskeletal pain, its impact on daily activities, discomfort in common body areas after padel play, and awareness of injury risks. Statistically significant differences were not observed between genders regarding the impact of pain on daily activities (χ^2^ = 1.558, *p* = 0.459). Both male and female players reported experiencing pain that sometimes affected their daily activities, with no significant trends noted for severity.

### 3.11. Awareness of Injury Risks

Awareness of injury risks did not show statistically significant gender differences (χ^2^ = 4.085, *p* = 0.130). Both male and female players demonstrated similar awareness levels, although some respondents indicated uncertainty (“maybe”) about injury risks associated with padel.

## 4. Discussion

This study highlights critical gender-specific differences in padel participation, injury patterns, and preventive practices among players in Madinah. Female players were found to have longer practice durations, higher rates of upper body injuries (particularly ligament sprains and muscle strains), and more frequent use of specialized equipment compared to male players. Male players exhibited distinct patterns of playing frequency and amateur-level participation, which influenced their injury risks. Stress, diet, and physical activity levels emerged as notable lifestyle factors contributing to injury prevalence.

The observed higher severity of injuries among female players can be attributed to several interconnected factors. First, the results showed that female players are more likely to participate at the intermediate and professional levels (Table 2), which involve higher intensity and competitive demands, potentially leading to increased injury risks. Second, the types of injuries reported—ligament sprains and muscle strains—are consistent with known biomechanical differences between genders. Women are more prone to ligamentous injuries due to hormonal effects, which may influence joint stability and neuromuscular control [17]. These findings suggest the need for gender-specific injury prevention strategies, such as targeted strength training and enhanced biomechanical education, particularly for players at advanced levels [8]. This conclusion is supported by the demographic disparities, which show that the duration of practice was much longer for the women in contrast to the male players; this implies increased exposure to motions that are repeated, possibly exacerbated through competitive contexts for a potential increase in injuries.

The overall injury prevalence and patterns observed align with findings in similar research. For example, ligament sprains and muscle strains are commonly reported in racket sports due to repetitive motions and biomechanical demands [5,6,7,8,9,10]. Upper limb injuries, particularly in the shoulder and wrist, were predominant among female players, consistent with Martín-Miguel et al. (2023), who attributed such injuries to repetitive overhead strokes and racket orientation [8]. However, the injury rates in this study were higher than those reported in Dahmen et al. (2023), who observed a lower incidence in recreational settings [9]. This discrepancy may reflect regional differences in playing conditions, training levels, or equipment use. The increased use of specialized footwear among female players may suggest a higher awareness of injury risks; however, this precaution alone appears insufficient to mitigate upper limb injuries. These findings highlight the need for a comprehensive approach that combines equipment optimization with training interventions.

The findings regarding lifestyle factors such as stress and physical activity levels corroborate the broader public health literature. High stress levels, cited by 94.8% of respondents, are known to impair musculoskeletal recovery and increase injury risk [16]. Similarly, poor dietary habits and low physical activity contribute to reduced physical resilience, as highlighted by Ding et al. (2022) [21]. Psychological pressures, such as performance expectations and intrinsic motivation, may influence injury risk and recovery among padel players. Previous studies have underscored the role of motivation and adherence in athletic performance and injury prevention [16]. Additionally, age-related and skill-based challenges in racket sports have been linked to psychological stress, further impacting players’ well-being and increasing their susceptibility to mental fatigue [19]. The strong association between stress levels and injury prevalence, as highlighted in the results, underscores the need for integrated psychological support programs. Such programs could address performance-related pressures while promoting resilience and adherence to preventive practices.

The observed gender differences underscore the need for tailored injury prevention strategies. Female players would benefit from targeted strength training programs to improve joint stability and reduce the risk of ligament sprains. Educational initiatives focusing on biomechanical challenges unique to female players, such as shoulder and wrist strain during overhead strokes, could further mitigate risks. Male players, on the other hand, would benefit from skill development programs aimed at improving technique and reducing amateur-level risks.

Structured interventions, such as warm-up programs and recovery strategies, could address the observed differences in injury patterns. For example, the results showed that men, who are less likely to use specialized equipment, may require greater emphasis on proper technique to reduce injury risks at amateur levels. Warm-up interventions, as recommended by Ding et al. (2022), have been shown to reduce injury rates by up to 36% [21]. Incorporating these programs into padel training sessions could significantly enhance player safety. Additionally, optimizing equipment, such as footwear quality and racket ergonomics, is essential to minimizing external risk factors.

These findings support the goals of Vision 2030 for increased sport participation and improvements in the health of the Saudi Arabian population. The findings have provided a framework for targeted health campaigns on injury prevention and lifestyle modification. A multisector collaboration between sports organizations, health providers, and policy makers could facilitate comprehensive strategies in injury prevention with an emphasis on local needs.

While this study provides valuable insights, it is limited by its cross-sectional design and reliance on self-reported data, which may introduce recall bias. The regional focus on Madinah limits the generalizability of findings to other populations. Future research should employ longitudinal designs to monitor injury trends over time and explore the efficacy of intervention programs. Moreover, the investigation into psychological factors, such as stress and motivation, may further extend the understanding of injury risk.

This study has one major limitation, which is its failure to take into consideration the external factors like court surface, weather, and environment which could greatly influence the risk of injuries among padel players. These factors have been found to contribute toward both acute and chronic injuries in wider research concerning sports injuries. Future studies should incorporate these variables into the design of their study so as to provide a broader perspective in comprehending the multifactorial causes of injury in padel.

One limitation of this study is the absence of effect sizes and confidence intervals in the analysis. While this study focused on categorical data and descriptive statistics, these metrics are valuable for interpreting the practical significance and precision of findings. Future studies should incorporate effect sizes and confidence intervals to provide a more comprehensive understanding of injury patterns and risks.

By addressing such limitations, any future research, based on the result of this study, will contribute to safer participation in padel and other similar emerging sports in a more inclusive way.

## 5. Conclusions

This study provides significant insights into the gender-specific injury patterns, participation trends, and lifestyle factors among padel players in Madinah. Key findings revealed that female players had higher injury rates, particularly upper limb injuries like ligament sprains and muscle strains, while male players displayed higher participation at the amateur level, exposing them to distinct injury risks. Stress, diet, and physical activity levels emerged as major contributing factors influencing injury prevalence across both genders.

The results emphasize the importance of tailored injury prevention strategies. Female players require targeted strength and conditioning programs to enhance joint stability, whereas male players would benefit from structured skill development and warm-up interventions to address technique-related injury risks. Moreover, improving equipment quality, particularly footwear, and fostering awareness about injury prevention can help minimize risks.

## Figures and Tables

**Table 1:** Characteristics of the participants.

Subjects	Female *n* (%) Mean ± SD	Male *n* (%) Mean ± SD	Total *n* (%) Mean ± SD	*p* Value
Age (years)	27.82 ± 6.04	24.36 ± 5.30	26.55 ± 6.01	0.008
How Long Practicing (months)	16.60 ± 8.21	10.75 ± 645	14.45 ± 8.10	0.000
How Long Playing (hours)	1.87 ± 0.57	1.35 ± 0.32	1.68 ± 0.55	0.188
Participants *n* (%)	193 (63.3%)	112 (36.7%)	305 (100%)	
Do You Use Special Shoes (yes)	127 (41.6%)	57 (18.7%)	184 (60.3%)	0.010
Satisfied with Madinah Padel Experience				
Yes	126 (41.3%)	64 (21.0%)	190 (62.3%)	0.239
Sometimes	45 (14.8%)	36 (11.8%)	81 (26.6%)	

**Table 2:** Padel game orientation factors.

Subjects	Female *N* (%)	Male *N* (%)	χ^2^ Value	*p* Value
How Many Times Playing Padel—Per week			49.424	0.000
1	42 (13.8%)	67 (22.0%)		
2	55 (18.0%)	22 (7.2%)		
3	55 (18.0%)	17 (5.6%)		
4	13 (4.3%)	4 (1.3%)		
5	12 (3.9%)	1 (0.3%)		
6	8 (2.6%)	0 (0.0%)		
7	8 (2.6%)	1 (0.3%)		
Professional Level in Padel Game			51.308	0.000
Amateur	43 (14.1%)	71 (23.3%)		
Intermediate	120 (39.3%)	34 (11.1%)		
Professional	30 (9.8%)	7 (2.3%)		
Have You Ever Suffered Any Injuries while Playing Padel?			65.915	0.000
Yes	136 (44.6%)	25 (8.2%)		
No	57 (18.7%)	87 (28.5%)		
What Is the Goal of Practicing?			20.720	0.079
Entertainment and Free Time	58 (19.0%)	55 (18.0%)		
Fitness and Health	46 (15.1%)	30 (9.8%)		
Improve Skills and Performance	42 (13.8%)	14 (4.6%)		
Participate in Tournaments and Challenges	35 (11.5%)	11 (3.6%)		
Others Reason	12 (3.9%)	2 (0.6%)		

**Table 3:** Comparison of injury and pain management while playing padel and after.

Subjects	Women *N* (%)	Men *N* (%)	χ^2^ Value	ρ Value
The Severity of the Injury			5.640	0.060
Minor	63 (39.1%)	17 (10.6%)		
Moderate	65 (40.4%)	7 (4.3%)		
Severe	9 (5.6%)	0 (0.0%)		
Injury Type			72.502	0.000
Ligament Sprain	31 (10.1%)	9 (3.0%)		
Meniscus	3 (1.0%)	1 (0.3%)		
Muscle Strain	37 (12.1%)	4 (1.3%)		
Tendinitis	19 (6.2%)	2 (0.7%)		
Dislocation	3 (1.0%)	1 (0.3%)		
Fracture	3 (1.0%)	0 (0.0%)		
Combination Injuries	26 (8.5%)	6 (1.2%)		
Where You Experience Pain—Most Common Area of Body			10.254	0.006
Lower	58 (19.7%)	15 (5.1%)		
Upper	124 (42.2%)	87 (29.6%)		
Both	7 (2.4%)	3 (1.0%)		
Musculoskeletal Pain Experience after Playing			16.841	0.001
Never	12 (3.9%)	23 (7.5%)		
Scarcely	76 (24.9%)	46 (15.1%)		
Sometimes	90 (29.5%)	35 (11.5%)		
Always	15 (4.9%)	8 (2.6%)		

**Table 4:** Impact of pain, injury, and awareness on padel players.

Particulars	Minor *n* (%)		Moderate *n* (%)		Severe *n* (%)		χ^2^ Value		ρ Value	
	m	f	m	f	m	f	m	f	m	f
Pain Affects Daily Activities							1.558	25.875	0.459	0.00
Yes	2 (8.3)	3 (2.2)	2 (8.3)	18 (13.1)	0 (0)	5 (3.6)				
Sometimes	9 (37.5)	21 (15.3)	3 (2.2)	28 (20.4)	0 (0)	2 (1.5)				
Awareness of Injury Risk							4.085	3.771	0.130	0.438
No	7 (29.2)	11 (8.0)	0 (0)	8 (5.8)	0 (0)	1 (0.7)				
Yes	6 (25.0)	29 (21.2)	4 (16.7)	40 (29.2)	0 (0)	4 (2.9)				
Maybe	4 (16.7)	23 (16.8)	3 (12.5)	17 (12.4)	0 (0)	4 (2.9)				

## Data Availability

The data presented in this study are available on request from the corresponding author.
